# Response of a tropical tree to non-timber forest products harvest and reduction in habitat size

**DOI:** 10.1371/journal.pone.0183964

**Published:** 2017-08-29

**Authors:** Orou G. Gaoue, M’Mouyohoun Kouagou, Armand K. Natta, Choukouratou Gado

**Affiliations:** 1 Department of Botany, University of Hawaii at Manoa, Honolulu, Honolulu, HI, United States of America; 2 Faculty of Agronomy, University of Parakou, Parakou, Benin; 3 Department of Geography, Environmental Management and Energy Studies, University of Johannesburg, APK Campus, Johannesburg, South Africa; Pacific Northwest National Laboratory, UNITED STATES

## Abstract

Non-timber forest products (NTFPs) are widely harvested by local people for their livelihood. Harvest often takes place in human disturbed ecosystems. However, our understanding of NTFPs harvesting impacts in fragmented habitats is limited. We assessed the impacts of fruit harvest, and reduction in habitat size on the population structures of *Pentadesma butyracea* Sabine (Clusiaceae) across two contrasting ecological regions (dry vs. moist) in Benin. In each region, we selected three populations for each of the three fruit harvesting intensities (low, medium and high). Harvesting intensities were estimated as the proportion of fruits harvested per population. *Pentadesma butyracea* is found in gallery forests along rivers and streams. We used the width of gallery forests as a measure of habitat size. We found negative effects of fruit harvest on seedling and adult density but no significant effect on population size class distribution in both ecological regions. The lack of significant effect of fruit harvest on population structure may be explained by the ability of *P*. *butyracea* to compensate for the negative effect of fruit harvesting by increasing clonal reproduction. Our results suggest that using tree density and population structure to assess the ecological impacts of harvesting clonal plants should be done with caution.

## Introduction

Wild plants are harvested worldwide for timber and non-timber forest products (NTFP) that serve as important sources of medicine, food and income for millions of people [1,2]. Unlike timber, NTFPs harvesting is expected to have limited impact on forests composition and structure. Hence, NTFPs harvest has been considered as a potential win-win solution to alleviate poverty while reducing the ecological impacts of resources extraction from the forest. However, the exploitation of NTFPs can have direct impacts on the survival, growth and reproduction of the exploited individuals, population structure and dynamics [1,3–5]. For example, harvesting *Catha edulis* and *Rapanea melanophloeos* for medicinal purpose has led to the reduction in the size of individuals and altered population structure in South Africa [6]. Similarly, populations of *Khaya senegalensis* harvested for their foliage and bark have reduced reproductive performance, population structure, and age at first reproduction which ultimately translated into reduced population growth rate [7,8]. Overexploitation of NTFPs such as fruits can limit seedling recruitment and affect population dynamics [3].

Understanding the biological consequences of harvesting and estimating sustainable harvest rate is central to developing sound forest management plans [9]. Several approaches have been used to assess the ecological impacts of NTFPs harvesting [1,10]. An increasing number of studies have used stage-structured matrix projection models for that purpose [5]. However, the widespread use of projection matrix models is limited by the requirement to collect data over several years (>3 y) to develop robust models. Several studies have used population size class distribution to evaluate the effect of NTFPs harvest [3,11–14]. Using population structure to assess the sustainability of harvest is based on the assumptions that harvested populations with negative exponential size class distribution are harvested sustainably. Harvested populations whose structure departs from this negative exponential distribution are deemed recruitment limited and therefore cannot maintain themselves. These assumptions are often inaccurate because the effect of NTFPs harvest on population structure is compounded by the life form and light tolerance of the species, habitats type, stand maturity and the ecological conditions in which harvesting is taking place [11].

Using population structure to assess the sustainability of NTFP harvesting may be further complicated by the ability of some plant species to allocate disproportionately more resources to clonal reproduction as a response to disturbance [15]. This shift in reproductive allocation can buffer the effect of NTFP harvest by increasing the proportion of clonal offspring thereby masking the effect of harvest on seedling recruitment and population dynamics. Although several plants reproduce both via seeds and clonal offspring, our understanding of the role of clonal reproduction on plant population response to NTFPs harvest is still limited. In addition, although harvesting NTFPs often takes place in a context of multiple stressors (fire, grazing; [16]) and most notably in increasingly fragmented and ecologically heterogeneous ecosystems, the synergistic effect of these drivers on population response to NTFP harvest is rarely studied [7,17–19]. In this paper, we investigate how a tropical tree species with both sexual and asexual reproduction responds to NTFP harvesting in habitats with varying size across ecological regions.

*Pentadesma butyracea* Sabine (Clusiaceae) is a multipurpose species with both clonal and sexual reproduction and found in the gallery forests in Benin. Populations of *P*. *butyracea* are threatened by overexploitation and habitat destruction [20–22]. Change in land use in our study region has led to an increasing conversion of gallery forests, a corridor of dense vegetation with fertile soils found alongside streams, into agricultural lands. As a result, the width of several gallery forest has shrunk under pressure from farms in neighboring savannas [21]. *Pentadesma butyracea* is also a multipurpose use tree and several organs such as the seeds, young shoots are overharvested [21]. The seeds are heavily harvested to make a butter [20]. This butter is often used as a substitute for the shea butter. Fruit harvesting is a major threat for *P*. *butyracea* since it can affect seedling recruitment [21]. We investigated if and how fruit harvest alters *P*. *butyracea* density, population structure and how such effect may vary with differences in habitat size and across ecological regions.

## Materials and methods

### Study areas

We studied populations of *P*. *butyracea* in the Republic of Benin (6°-12°50’ N; 1°-3°40’ E), West Africa. Benin is part of the Dahomey Gap, a dry corridor that split the African rain forest into two blocks [23]. Thus, the country is drier than neighbouring countries such as Nigeria and further west, Ghana. The mean annual rainfall varies between 900 (Sudanian region) and 1300 mm (Guineo-Congolian region). The vegetation is dominated by savanna with gallery forests along waterways in the Sudanian (9°30’-12°N) and Sudano-Guinean (7°30’-9°30 N) regions and by agricultural lands, remnant Guinean savanna with small patches of semi-deciduous forests and sacred groves in the Guineo-Congolian region (6°-7°30 N). There is an ecological gradient from the southern Guineo-Congolian region to the northern Sudanian region [24]. The Sudano-Guinean region with annual rainfall of 1100–1300 mm and the annual temperature of 25–29°C and less mean annual insolation (2420 h) is wetter than the Sudanian region with mean annual rainfall, temperature and insolation of 900–1100 mm, 24°C–31°C and 2660 h respectively [25]. The Sudanian region also has longer dry season (7 months) than the Sudano-Guinean region (6 months). Our study species, *P*. *butyracea*, is found in the Sudano-Guinean and Sudanian regions [20] and we selected our study populations along this ecological gradient.

### Study species

*Pentadesma butyracea* (Clusiaceae) or tallow tree is a dense forest tree widely distributed in Africa from Guinea-Bissau to the West of the Democratic Republic of Congo [21,26]. The tree can reach up to 20–35 m in height and 80–100 cm diameter at breast height (dbh). It produces hermaphrodite flowers that are self-incompatible; the fruits are 12.9 ± 3.2 cm long and 8.6 ± 2.cm wide berries which and contain 1 to 34 seeds inside a yellow mesocarp [26]. *Pentadesma butyracea* reproduces both via seeds and clonally. In populations that are heavily harvested for their fruits, the proportion of recruits represented by clonal offspring can reach up to 95% which represent a 30% increase from low harvested populations [27]. In Benin, *P*. *butyracea* is found in gallery forests. Gallery forests are habitat for nearly 30% of Benin’s flora with 129 to 358 species ha^-1^ and represent important refugia for rare and endangered species [28]. However they are threatened worldwide by agricultural expansion and climate change [29]. This dense vegetation is surrounded by savanna with a sharp ecotone that makes it easy to estimate the width of the gallery. The width of these gallery forests often varies depending upon the size of the rivers or the impacts of anthropogenic activities.

### Estimating fruit harvesting rate and gallery forest width

We sampled populations of *P*. *butyracea* in gallery forests in the Sudano-Guinean and Sudanian regions of Benin. From 2007 to 2012, we identified a total of 31 populations of *P*. *butyracea* in Sudanian region and 74 populations in Sudano-Guinean region. The distribution of *P*. *butyracea* is limited to three main areas in the Northwestern part of the country [30] and this made it easier to survey the populations. We asked field forestry officers and local farmers of known populations of *P*. *butyracea*. We then visited these sites to confirm the presence of the study species. No specific permissions were required for the data collection because the study species is not endangered or protected and our populations were sampled outside of protected areas. In each of the two ecological regions, we randomly selected nine populations equally distributed among three fruit harvesting intensities: low (< 25% fruits harvested), medium (25–75% fruits harvested), and high (≥ 75% fruits harvested). These populations were distributed across four latitudes and separated by a minimum of 30km. The total density of individuals that have reached reproductive size (dbh >10cm) ranged from 80 to 380 trees/ha. For each of the 18 populations, we measured the width of the gallery forest as a metric of *P*. *butyracea* habitat size. Gallery forests width ranged from 13.3 m to 80 m and was measured perpendicularly from the riverbed to the edge of the neighboring savanna. In each population, we established three rectangular plots of 500m^2^ each to sample *P*. *butyracea* individual with diameter at breast height (dbh) ≥10 cm. The dimensions of the plot were constrained by the width of the gallery forest. In each plot, five 5 × 5-m quadrats were installed to sample individuals with dbh < 10 cm. For each individual, we measured dbh, height and for each plot, we estimated tree density.

To confirm harvesting histories, we conducted semi-structured interviews with 90 women living around the studied populations and who harvest these fruits. Harvesting has been taking place in these populations for decades and in our semi-structured interviews, we confirmed that sites that were identified as moderately or heavily harvested had been harvested similarly over the past decade. Therefore, although fruit harvest intensity was estimated in one year, this captures the long-term harvesting histories of the populations we sampled. In most cases, there were evident reasons why some populations were less harvested than others. Low or unharvested populations were located in protected areas (forest reserves and State forests) and in remote places that are difficult to access by women (harvesters) and therefore remain unharvested for years. None of the medium-harvest populations was in a protected area. However, they were located in remote areas where only those whose farms were in the areas could harvest these populations. Heavily harvested populations were closer to villages and therefore experienced high fruit-harvesting pressure.

We estimated fruit harvesting intensity as the proportion of total fruit produced that is removed from the population. For each population, we first estimated the number of fruits produced per reproducing trees by visually counting fruits on each *P*. *butyracea* tree during the fruiting period (March-May 2012). *P*. *butyracea* trees were 4–28 m tall in the populations we studied. However, the branches are spread and the fruits are large (1–29 cm length and 2.8–15 cm diameter) and yellowish which made it easier to see the fruits and count them visually. For each tree, two field assistants separately counted the number of fruits and we averaged the number of fruits they obtained for each tree. We then estimated the number of fruits that had fallen on the ground but that were intact (not collected by harvesters). Harvesters process the fruits at the place where they collected them. Given that they are interested in the seeds rather than the fruits, harvesters remove the seeds and leave the mesocarp (pulp) next to the reproducing *P*. *butyracea* trees. This has allowed us to estimate the number of fruits that was collected out of the total number of fruits produced (fruits on trees and intact fruits on forest floor) in a given population. We estimated fruit harvesting intensities for each population as the ratio between the number of fruits collected and the total number of fruits in that population. We assumed no frugivory because the fruits are large and are less likely to have been removed from the populations. Elephants and monkeys are known to consume and disperse fruits of *P*. *butyracea* [31]. However, most savanna elephant populations in Benin and elsewhere in the world are threatened by poaching and limited to the National Parks [32–34] which are not included in this study. This limit their dispersal ability for several plant species including *P*. *butyracea* [31]. Similarly several Cercopithecus monkeys are threatened by hunting and habitat loss [35,36] in our study populations. We expect that if there was frugivory by these known frugivores, this will be limited and less likely to significantly affect our estimate of *P*. *butyracea* fruit number.

### Data analysis

To test the effect of fruit harvest, ecological zone and gallery forest width on seedling and adult densities, we used glmmADMB package in R 3.1.2. to fit a generalized mixed-effects models (GLMM) with a negative binomial error structure (to control for overdispersion), with population as random effect and harvesting intensity, ecological region and gallery forest width as fixed effects including the interactions between these fixed effects. In all of our models we standardized the continuous variable (gallery forest width) as suggested by [37]. We tested eight different nested models and used an information-theoretic approach [38] to estimate models’ Akaike Information Criterion (AIC), and ΔAIC, the difference between the AIC of each model *i* and the AIC for the model with the lowest AIC value. To estimate the effect of predictors on seedling or adult density, we use package ‘MuMin’ for model averaging [39]. Model averaging allows us to include all the eight candidate models and account for model selection uncertainty while ensuring robust estimate of regression coefficients [40].

We used a conditional log-linear analysis to test the effect of fruit harvesting rate and ecological difference between regions on tree diameter class distribution. We selected five dbh size classes with 10-cm increment. Fruit harvesting rate, ecological region and diameter class were considered as independent variables and the number of individuals in each diameter class as the dependent variable. We used ΔAIC to select the best-fitting models. All statistical analyses were conducted in R [41] and all data on *P*. *butyracea* demography are available from the Dryad Digital Repository [42].

## Results

**Fig 1:**
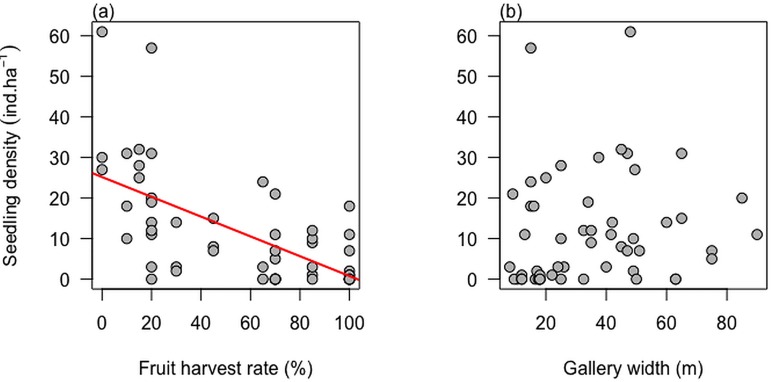
Effect of fruit harvesting rate (a) and gallery forest width (b) on *Pentadesma butyracea* seedling density.

**Fig 2:**
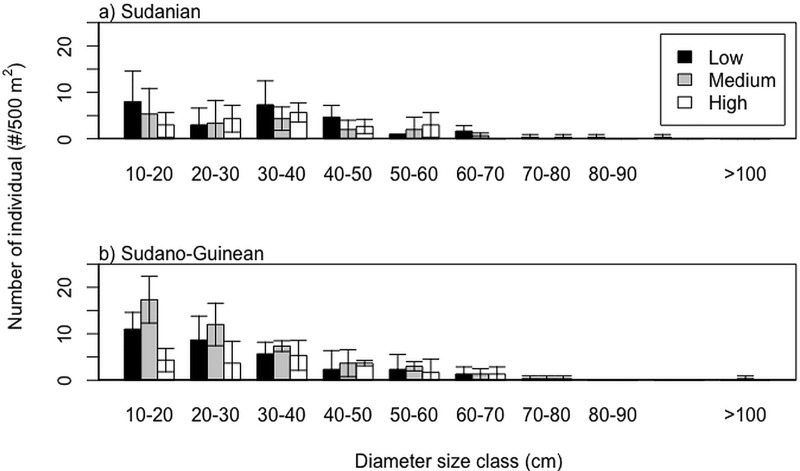
Size class distribution for *Pentadesma butyracea* populations with low, medium and high fruit harvesting rates in two ecological regions.

We found significant effect of fruit harvest (Fig 1A) but no effect of habitat size (Fig 1B) on seedling and adult density (Tables 1 and 2). Seedling density was higher in low than high harvest populations (Table 1: β_Low v. High_ = 0.65 ± 0.27; Z = 2.40, P = 0.0162). Seedling density was higher in medium harvest populations than in high harvest populations (β_Medium v. High_ = 0.44 ± 0.27, Z = 1.65, P = 0.098). In addition, we found no significant effect of region although higher seedling density was observed in the moist Sudano-Guinean than in the dry Sudanian region (β_S v. SG_ = 0.44 ± 0.24, Z = 1.882, P = 0.067). Similarly to seedlings, adult density was significantly higher in low-harvest populations than in medium- or high-harvest populations (Table 2: β_Low v. High_ = 0.58 ± 0.26, Z = 2.23, P = 0.025). However, there was no difference in adult density between ecological regions or as a function of gallery forest width (Table 2). Similarly, we found no significant effect of fruit harvest on size class distribution (Fig 2; Table 3).

**Table 1:** Effect of fruit harvest (Harvest: L = low, M = medium, H = high), gallery forest width (FSize) on *Pentadesma butyracea* seedlings density in two ecological regions of Benin (RegSG vs. RegS). Regression coefficients, adjusted 1 SE and P-values are estimated from information theoretic model averaging approach following a generalized mixed effect model with negative binomial error structure.

Predictors	Estimates	Adjusted SE	z	P
Intercept	3.826	0.290	13.213	<0.0001 ***
Reg SG	0.438	0.239	1.832	0.0670
Harvest L	0.650	0.270	2.404	0.0162 *
Harvest M	0.438	0.265	1.651	0.0988
FSize	0.054	0.284	0.189	0.8498
Harvest L × FSize	-0.402	0.344	1.166	0.2435
Harvest M × FSize	-0.491	0.338	1.454	0.1459
FSize × RegSG	-0.367	0.319	1.15	0.2501
Harvest L × RegSG	0.057	0.671	0.085	0.9322
Harvest M × RegSG	0.335	0.830	0.404	0.6861
Harvest L × FSize × RegSG	-0.181	0.932	0.194	0.8459
Harvest M × FSize × RegSG	-0.418	1.247	0.335	0.7373

* P < 0.05

*** P < 0.001.

**Table 2:** Effect of fruit harvest (Harvest: L = low, M = medium, H = high), gallery forest width (FSize) on *Pentadesma butyracea* adult density in two ecological regions (RegSG vs. RegS). Regression coefficients, adjusted 1 standard error (SE) and p-values are estimated from information theoretic model averaging approach following a generalized mixed effect model with negative binomial error structure.

Predictors	Estimates	Adjusted SE	z	P
Intercept	1.738	0.321	5.406	<0.0001 ***
Reg SG	0.321	0.257	1.252	0.2107
Harvest L	0.584	0.262	2.232	0.0256 *
Harvest M	0.653	0.437	1.495	0.135
FSize	-0.091	0.401	0.226	0.8211
FSize × RegSG	0.346	0.485	0.713	0.4758
Harvest L × FSize	0.030	0.453	0.067	0.9466
Harvest M × FSize	0.515	0.578	0.892	0.3723
Harvest L × RegSG	0.443	0.489	0.907	0.3645
Harvest M × RegSG	1.169	0.815	1.434	0.1515
Harvest L × FSize × RegSG	-0.573	0.596	0.962	0.336
Harvest M x FSize × RegSG	-0.615	0.764	0.805	0.4209

* P < 0.05

*** P < 0.001.

**Table 3:** Model selection for the effect of region (*Reg*) and fruit harvesting rate (*Harvest*) on the size class distribution (*SClass*) of *Pentadesma butyracea* populations. Each model includes a set of main effects of region, harvest and size class (*no effect* model). Δ_*i*_, the difference between the smallest Akaike Information criterion (AIC) value and the AIC for each candidate model was used to select the best model. Models with Δ_*i*_*<*2 were considered best supported.

Model definition	Model	AIC	Δ_*i*_
No effect	Reg + Harvest + SClass	293.42	**0**
Harvesting effect	Harvest × SClass	303.18	9.76
Region effect	Reg × SClass		
Additive effect of Harvest and Region	Harvest × SClass + Reg × SClass	306.5	13.08
Interactive effect of Harvest and region	Reg × Harvest × SClass	322.04	28.62

## Discussion

Reduction in habitat size as a result of forest fragmentation can affect plant population biology [43–47]. Non-timber forest product harvesting can also reduce plant population dynamics [1,3,7,48,49]. However, understanding of the synergistic effect of NTFPs harvesting and habitat fragmentation is limited. With increasing level of ecosystem fragmentation across the world, most NTFPs harvesting will likely take place in habitat that are experiencing multiple stressors including more frequent fire, changes in climate patterns, and reduction in land cover areas due to changes in land use strategies [50,51]. We studied the effects of fruit harvest by local people from a tropical clonal tree and the reduction in its habitat size on its population density and structure across ecological regions.

We found negative effects of harvest on seedling and adult density but no significant effect of fruit harvest on *P*. *butyracea* population structure across ecological regions and regardless of gallery forest width. Nevertheless, medium-sized trees with (dbh < 30 cm) were scarce in the dry Sudanian zone where the gallery forests sustain more degradation and often converted into farms. Regardless of the ecological zone or fruits harvesting intensity, larger trees (dbh > 70 cm) were scarce and this may be due to size selective illegal logging of these individuals in our study areas [21]. This result was robust to changes in habitat size (width of gallery forest).

The lack of significant effect of fruit harvest on size class distributions in a clonal plant such as *P*. *butyracea* should be taken with caution. First although size class distributions in harvested versus unharvested populations are similar and negative exponential, it does not necessarily suggest that there is no effect on population dynamics. For example, there was no significant effect of foliage and bark harvesting on the population structure of *Khaya senegalensis* in the moist regions of Benin [52] but using matrix projection modelling, the authors were able to show significant effects of harvest on the population dynamics of the species in that same region [7,53,54]. Consistent with this, previous studies show that there is no strong link between population structure and dynamics [55,56]. Second for species such as *P*. *butyracea* which have dual sexual and asexual reproductive capacity, there is a possibility that they compensate for loss of seed sources, via fruit harvesting, by increasing clonal output which may buffer the negative effect of harvest on seeding recruitment and population structure. Such compensatory mechanisms can also be triggered or reinforced by other type of disturbance that often follow NTFP harvesting such as fire, trampling or random cutting of neighboring plants [57]. Consistent with this assumption, the highest regeneration was observed in the Setou (15040 stems ha^-1^) and Tchoundékou (14160 stems ha^-1^) populations which were severely affected by fire but were moderately harvested.

Previous studies on the impacts of NTFPs harvest on plant population structure and dynamics yield mixed results. Some studies reported significant structural changes in harvested populations. Several studies showed that NTFPs harvest has significant effect on size class distribution for species harvested for organs other than fruits (e.g. foliage, roots or bark) [1,6,11]. Harvesting bark from *Waburgia salutaris* [6], *Anogeissus leiocarpa* [13], bark and leaves from *Adansonia digitata* [58] and *Khaya senegalensis* [11] or harvesting resins from *Canarium strictum* [59] significantly affected population structure. In general, most studies on the effects of fruits or seeds harvest did not report significant harvest-related changes in size class distributions [12,60,61]. However, there were significant differences in the size class distributions between harvested and unharvested population for our study species [20]. This study was conducted in a limited regional scale and included a smaller number of independent populations. Failing to account for the between populations ecological variation can affect understanding of the effect of NTFPs harvesting on population ecology [1,53].

Chronic nut harvesting on *Bertholletia excelsa* population may negatively affect size class distribution [3]. The fact that this study has documented the effects of long-term harvesting suggest that the time scale on which most studies, including ours, have tested the effect of fruit harvesting on population structure is not adequate. However, some kind of fruit harvesting can have demographic effect if it leads to branch removal like in the case of Amla in India [62,63]. In that case, the effect would be due to branch cutting rather than fruit harvest. It has been demonstrated that recurrent cutting of branches can have significant and far-reaching ecological effects [4,54,64]. This kind of harvesting may affect population structure by delaying growth and progression in larger size classes.

## Conclusions

In this study, we show that recurrent harvesting of fruits from a tropical tree, *Pentadesma butyracea*, has significant effect on seedling and adult densities but no significant effect on population structure regardless of habitat size. This lack of effect can be due to the delay between the limitation of seed sources and its effects on the transition of individuals through plant life cycle. We suggest that an increase in clonal reproduction, such the one observed in our study system [27], can potentially buffer the short-term effect of seeds loss, via fruit harvesting. Heavily harvested populations of *P*. *butyracea* have 30% more clonal offspring than low harvested populations. However, future work on similar system should investigate how such lack of significant effects on population structure translates into effect or lack thereof on long-term population dynamics. Since *P*. *butyracea* is harvested for multiple purposes (fruits, bark harvest. cuts young stems for toothbrush, harvest of young leaves and logging), it is critical to disentangle the main or interactive effects of these stressors on population dynamics.
